# Successful hepatectomy for hepatic abscess with chronic granulomatous disease: a case report

**DOI:** 10.1186/s40792-017-0333-z

**Published:** 2017-04-26

**Authors:** Ryo Muranushi, Makoto Suzuki, Kenichiro Araki, Norio Kubo, Sayaka Otake, Yutaka Nishida, Takashi Ishige, Hirokazu Arakawa, Hiroyuki Kuwano, Ken Shirabe

**Affiliations:** 1 Department of Hepatobiliary and Pancreatic Surgery, Gunma University Graduate School of Medicine, Gunma University, 3-39-33, Showa-Machi, Maebashi, Gunma 371-8511 Japan; 2 Division of Pediatric Surgery, Integrative Center of Surgery, Gunma University Hospital, Gunma, Japan; 3 Department of Pediatrics, Gunma University Graduate School of Medicine, Gunma University, Gunma, Japan; 4 Department of General Surgical Science, Gunma University Graduate School of Medicine, Gunma University, Gunma, Japan

**Keywords:** Hepatectomy, Hepatic abscess, Chronic granulomatous disease

## Abstract

**Background:**

Chronic granulomatous disease (CGD), a rare inherited disorder, is characterized by impaired ability of phagocytic cells to kill certain bacteria and fungi. Although liver abscess is a common manifestation of CGD, its optimal management in these patients is unknown. Here, we present a case of successful hepatectomy for hepatic abscess in a patient with CGD.

**Case presentation:**

An adolescent patient with previously diagnosed CGD presented to the pediatrics department of our institution with fever. Blood tests showed high concentrations of inflammatory markers. A computed tomography (CT) scan showed a multilocular mass measuring 52 mm × 34 mm in hepatic segment 4 (S4). Blood cultures were negative. Despite administration of antibiotics and *γ*-globulin, his fever and high concentrations of inflammatory markers persisted and the mass did not change on CT scan images. Because the medications had proved ineffective and percutaneous drainage would have been difficult because of the honeycombing in the abscess, we performed hepatic S4a + S5 anatomic resection and cholecystectomy. Culture of the excised specimen was negative. The patient’s postoperative course was uneventful. On day 62, CT showed no abscess around the resection stump. On day 81, he was transferred to undergo bone marrow transplantation.

**Conclusions:**

Surgical treatment for hepatic abscess can be effective when medical treatment has failed.

## Background

Chronic granulomatous disease (CGD), a form of inherited primary immunodeficiency (PID), is characterized by the inability of phagocytes to produce reactive oxygen intermediates because of a defect in the nicotinamide adenine dinucleotide phosphate (NADPH) oxidase complex. Five distinct genetic forms affect the components of nicotinamide adenine dinucleotide phosphate oxidase—gp91^*phox*^, p22^*phox*^, p47^*phox*^, p67^*phox*^, and p40^*phox*^—resulting in diminished phagocyte respiratory bursts [1]. Patients with CGD characteristically have recurrent and life-threatening infections. Although prophylactic antibiotics, antifungals, and interferon (IFN)-γ have improved outcomes, bone marrow transplantation (BMT) is the only curative treatment identified thus far. It is important to control infections prior to performing BMT.

Although hepatic abscess is a common manifestation of this disease, its optimal management has not been clearly defined in published reports. Additionally, general guidelines for treating pyogenic hepatic abscesses do not necessarily apply in patients with CGD. Surgery can reportedly be effective; however, careful consideration of surgical treatment is necessary because of its invasiveness and high rate of complications [2, 3]. Here, we present a case of successful hepatectomy for hepatic abscess in a patient with CGD.

## Case presentation

**Fig. 1:**
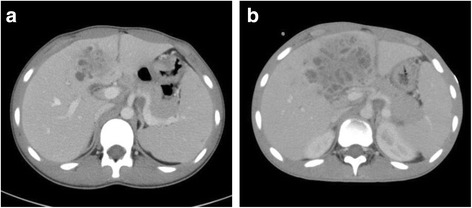
**a** Enhanced computed tomography (CT) image. CT image showing a multilocular mass measuring 52 mm × 34 mm in hepatic segment 4a and 5 (S4a + 5). **b** CT findings on day 28. The abscess has not changed since the previous scan and exhibits honeycombing

This adolescent patient had been diagnosed as having CGD with repeated pneumonia at 10 years of age and been followed up by the pediatrics department of our hospital. He was receiving prophylactic trimethoprim–sulfamethoxazole and minomycin and had received IFN-γ. At age 15 years old, he presented to the pediatrics department with fever. On physical examination, the abdomen was soft and flat with epigastric tenderness and there were old and new patches of impetigo on the trunk and limbs. Blood tests showed white blood cell count 9.0 × 10^9^/L (normal range, 3.0 × 10^9^ to 9.0 × 10^9^/L), C-reactive protein (CRP) 15.52 mg/dL (normal range, 0 to 0.3 mg/dL), procalcitonin (PCT) 0.78 ng/mL (normal range, 0 to 0.3 ng/mL), aspartate aminotransferase 18 U/L (normal range, 5 to 30 U/L), alanine phosphatase 16 U/L (normal range, 10 to 30 U/L), alkaline phosphatase 527 U/L (normal range, 100 to 350 U/L), total bilirubin 1.0 mg/dL (normal range, 0.2 to 1.2 mg/dL), and prothrombin time 56% (normal range, 85 to 125%). Computed tomography (CT) showed a multilocular mass measuring 52 mm × 34 mm in hepatic segment 4a and 5 (S4a + 5) (Fig. 1a). Blood cultures were negative. We considered performing a liver biopsy to identify the pathogenic bacteria. However, because previous studies have shown that cultures of hepatic abscesses in patients with CGD are often negative and percutaneous drains seem to be a focus of secondary liver infection, we did not perform a liver biopsy considering his immunologically deficient state [2, 4]. Meropenem, vancomycin, and voriconazole were administered, but his remittent fever and high concentrations of inflammatory markers (CRP 15.52 mg/dL, PCT 0.78 ng/mL) persisted.

On day 20 after admission, a CT scan showed the mass had enlarged, now measuring 83 mm × 59 mm; accordingly, *γ*-globulin was administered and amphotericin B substituted for voriconazole. However, his fever and high concentrations of inflammatory markers (CRP 15.52 mg/dL, PCT 0.78 ng/mL) persisted, and on day 28, the mass had not changed on CT imaging (Fig. 1b). Because the medications had proved ineffective and percutaneous drainage would have been difficult because of the honeycombing in the abscess, surgical treatment for his liver abscess was considered. Preoperative examinations showed an indocyanine green retention rate of 15 min (ICG R15) 3.8%, Child–Pugh grade A, liver damage grade A, and estimated remnant liver volume after hepatectomy 1799 mL (94.0%). Given that sufficient hepatic reserve could be preserved, a decision was made to perform a hepatectomy. Accordingly, on day 30, we performed anatomic resection and cholecystectomy.

**Fig. 2:**
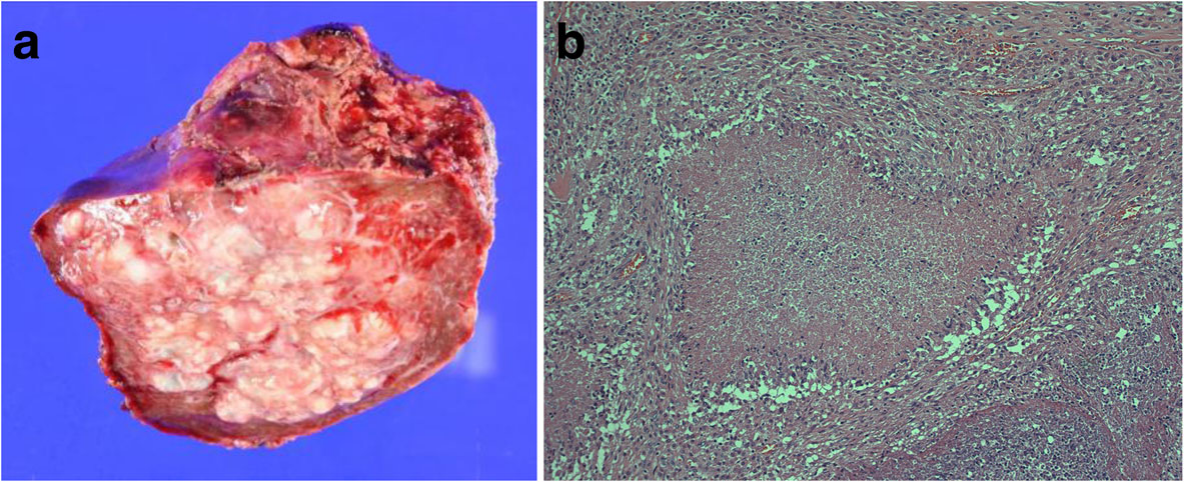
**a** Photograph of resected specimen. The resected liver weighed 290 g. The abscess was multiloculated and full of light green, malodorous, and viscous pus. **b** Histopathological findings. Histopathological examination showed epithelioid cell granuloma with caseous necrosis consistent with the diagnosis of CGD (H&E staining, ×100)

Broad, inflammation-related adhesions were found around the liver, which was enlarged, and a solid mass was palpated in S4. The resection line was identified with intraoperative ultrasound guidance and S4a + S5 anatomic resection performed. The resected liver weighed 290 g (Fig. 2a). The abscess was multiloculated and full of light green, malodorous, and viscous pus, culture of which was negative. Histopathological examination showed epithelioid cell granuloma with caseous necrosis, which is consistent with the diagnosis of CGD (Fig. 2b). Postoperatively, meropenem and voriconazole were administered. On postoperative day 4, he started oral ingestion, and on day 5, the percutaneous drain was removed. On day 20, inguinal lymphadenitis was noted and managed by incision and drainage. Subsequently, although mildly increased concentrations of inflammatory markers persisted, they improved with addition of vancomycin. On day 62, a CT scan showed no abscess around the resection stump, and on day 81, he was transferred to undergo bone marrow transplantation.

## Discussion

Abnormalities in numbers or function of phagocytes account for 20% of cases of PID. CGD is the most frequent of the phagocytic disorders, its prevalence being 1/220,000. In patients with CGD, the functional activity of NADPH oxidase is significantly diminished or completely absent, resulting in no production of superoxide derivatives. Affected patients have recurrent infections involving the lymph nodes, lungs, soft tissue, and liver, the causative organisms being catalase-positive bacteria and fungi such as *Staphylococcus aureus*, *Klebsiella pneumoniae*, *Escherichia coli*, *Aspergillus* species, and *Candida* species, which cannot be killed because of the absence of reactive oxygen [4].

The other clinical characteristic of CGD is granuloma formation associated with excessive inflammation; these can form in the gastrointestinal tract, lung, and genitourinary organs. Such granulomas are believed to form as a result of persistent activation of intracellular inflammatory signals by reactions to microorganisms, production of inflammatory cytokines because apoptotic calls are not eliminated, and persistence of activated inflammasomes attributable to abnormal autophagy [5–8].

Hepatic abscesses develop in 14–51% of patients with CGD [2, 9]. More than half of them are due to *S. aureus*; *Serratia* spp. and *Nocardia* spp. are also frequently isolated [10]. However, cultures of hepatic abscesses are often negative and blood cultures are rarely positive, resulting in resistance to antimicrobials [2].

There is a high rate of recurrence of hepatic abscesses in patients with CGD [9]. Hepatic dysfunction with portal venopathy and nodular regenerative hyperplasia (NRH) is reportedly strongly associated with liver abscesses and is a major predictor of mortality in patients with CGD [11]. The mechanism is presumed to be noncirrhotic portal hypertension caused by microvascular insults from repeated abscesses [11]. It is possible that hepatic regeneration after surgery causes venopathy and NRH [12].

Although there are a few published case reports and retrospective discussions of treatment of hepatic abscesses in patients with CGD, their management is not well-defined. Corticosteroids or long-term administration of antibiotics are reportedly effective forms of medical treatment [4]. Corticosteroids are considered effective against the granuloma formation that characterizes CGD. In the present case, however, because the presence of active infection was not ruled out, we avoided corticosteroids because they may have masked or favored dissemination of the pathogen. The patient’s inflammatory findings improved postoperatively; therefore, corticosteroids were unnecessary.

However, many cases are resistant to these approaches because of the difficulty in detecting pathogenic bacteria and characteristic granuloma formation, as mentioned previously. Consistent with this, in our case, blood cultures and culture of the resected specimen were negative. We believe that the failure in medical treatment was attributable to the hepatic abscess being aseptic.

To our knowledge, this is the first reported case of successful hepatectomy for hepatic abscess in a patient with CGD in Japan. Previous cases of CGD successfully treated by surgery were reported in the English language literature. Some researchers have recommended surgical treatment. Roback et al. in 1971 outlined treatment options gathered from their management of 6 hepatic abscesses in 25 patients with CGD [13]. They recommended partial hepatic lobectomy for peripheral abscesses and full and extensive debridement for central abscesses. Mouy et al. managed 14 hepatic abscesses in 48 patients with CGD over a 16-year period. Nine cases required operation; 2 were managed successfully with percutaneous drainage [14]. Lublin et al. reported the efficacy of surgery and percutaneous drainage in a retrospective study of 61 cases of hepatic abscess in patients with CGD [2]. These authors pointed out that pyogenic abscesses are generally filled with homogenous purulent material, whereas abscesses in patients with CGD are characteristically dense, septated masses with fibrous pseudocapsules containing thick, inspissated fluid. Because abscesses in patients with CGD can rarely be treated by percutaneous drainage, they recommended aggressive surgical management [2]. Chen et al. emphasized that primary hepatic resection is preferable to percutaneous drainage because it can be curative and is associated with shorter hospitalization [3].

However, the complication rate is higher in standard hepatic surgery because these patients are immunodeficient. The complication rate is reportedly 50–56%, complications including persistent hepatic abscess, wound dehiscence, pancreatitis, and pneumonia [2]. In patients who develop wound dehiscence, steroids are paradoxically effective because the slow wound healing is considered to be related to granuloma formation [2].

Based on the above findings, hepatectomy is considered appropriate for patients in whom medical treatment and percutaneous drainage have failed or are contraindicated, the abscess is surgically resectable, and a hepatic reserve can be ensured. Assessment of hepatic function is particularly important because transplantation may be necessary due to the progression of hepatic fibrosis or repeated bouts of inflammation.

Because peritonitis may occur by spillage of the purulent content during hepatectomy for liver abscesses, it is important to perform en bloc resection without perforation of the abscess. Although dissecting along the pseudocapsule plane effectively minimizes injury to major vessels, the most appropriate method must be determined by the location of the abscess. For deep abscesses, the liver can be split along its intersegmental planes to expose the pseudocapsule for enucleation. Preservation of the hepatic parenchyma is especially important in patients with CGD because of the high frequency of abscess recurrence. This minimizes the loss of hepatic parenchyma and leaves the vascular anatomy intact for future excisions. Additionally, minimal hepatic resection rather than major hepatectomy may be sufficient to control these abscesses while likely minimizing postoperative complications. Furthermore, adequate perioperative management is required.

In the present case, because the abscess was close to the root of Glisson’s sheath of S4a and S5, we performed S4a + 5 anatomical hepatic resection instead of limited nonanatomic resection to ensure that complete resection was achieved. Additionally, because it was necessary to resect the liver while exposing Glisson’s sheath from the first ramification to the anterior segment, we selected laparotomy instead of laparoscopic surgery in consideration of the complexity of the surgical procedure. We plan to apply a laparoscopic approach for peripheral small lesions in the future. In the present case, the abscess was completely resected by this procedure.

In our case, hepatectomy succeeded in controlling a hepatic abscess in a patient with CGD and enabling him to undergo BMT. It is important to control infection before subjecting patients with CGD to this radical treatment. Surgery should be carefully considered in patients whose abscesses prove resistant to medical treatment.

## Conclusions

We herein present a case of successful hepatectomy for a hepatic abscess in a patient with CGD. Surgical treatment for hepatic abscess can be effective when medical treatment has failed.

## Abbreviations

BMT: Bone marrow transplantation
CGD: Chronic granulomatous disease
CRP: C-reactive protein
CT: Computed tomography
IFN-γ: γ-interferon
NADPH: Nicotinamide adenine dinucleotide phosphate
NRH: Nodular regenerative hyperplasia
PCT: Procalcitonin
PID: Primary immunodeficiency
